# Economic evaluation of interventions to address undernutrition: a systematic review

**DOI:** 10.1093/heapol/czaa149

**Published:** 2020-12-06

**Authors:** Francesco Ramponi, Wiktoria Tafesse, Susan Griffin

**Affiliations:** Centre for Health Economics, Alcuin A Block, University of York ,York YO10 5DD, UK; Centre for Health Economics, Alcuin A Block, University of York ,York YO10 5DD, UK; Centre for Health Economics, Alcuin A Block, University of York ,York YO10 5DD, UK

**Keywords:** Decision-making, economic evaluation, systematic reviews, priority setting, cost-effectiveness analysis, cost–benefit analysis, maternal and child health, nutrition

## Abstract

Strategies to address undernutrition in low- and middle-income countries (LMICs) include various interventions implemented through different sectors of the economy. Our aim is to provide an overview of published economic evaluations of such interventions and to compare and contrast evaluations of interventions in different areas. We reviewed economic evaluations of nutrition interventions in LMICs published since 2015 and/or included in the Tufts Global registry or Disease Control Priorities 3rd edition. We categorized the studies by intervention type (preventive; therapeutic; fortification; delivery platforms), nutritional deficiency addressed and characteristics of the economic evaluation (e.g. type of model, costs and outcomes included). Of the 62 economic evaluations identified, 56 (90%) were cost-effectiveness analyses. Twenty-two (36%) evaluations investigated fortification and 23 (37%) preventive interventions. Forty-three percent of the evaluations of preventive interventions did not include a model, whereas most of fortification strategies used the same reference model. We identified different trends in cost categories and inclusion of health and non-health outcomes across evaluations in the four different topic areas. To illustrate the implications of such trends for decision-making, we compared a set of studies evaluating alternative strategies to combat zinc deficiency. We showed that the use of ‘off-the-shelf’ models and tools can potentially conceal what outcomes and costs and value judgements are used. Comparing interventions across different areas is fundamental to assist decision-makers in developing their nutrition strategy. Systematic differences in the economic evaluations of interventions delivered within and outside the health sector can undermine the ability to prioritize alternative nutrition strategies.

KEY MESSAGES

- Strategies to address undernutrition in low- and middle-income countries include various interventions implemented through different sectors of the economy. Comparing interventions across different areas is fundamental to assist decision-makers in developing a cost-effective nutrition strategy.
- Due to different frameworks for economic evaluation and differences in the costs and outcomes included for interventions delivered in different sectors, studies can be difficult to compare. For example, economic evaluations of interventions delivered within and outside the health system (e.g. nutritional supplements for malnutrition vs fortification of oil) often differ in the set of costs they include.
- The use of ‘off-the-shelf’ models and tools can conceal what outcomes, costs and value judgements are included in the economic evaluation. Our study suggests to highlight these underlying components in economic evaluations to promote the consistency and ease of interpretation.

## Introduction

Undernutrition relates to the inadequate intake of energy or vital nutrients, which leads to ill health effects that manifest in: stunting (i.e. low height-for-age); wasting (i.e. low weight-for-height); underweight (i.e. low weight-for-age); and micronutrient-related deficiencies (i.e. inadequacies in intake of vitamins and minerals) (WHO, 2018). Undernutrition in children leads to an increased risk of death and has been considered a leading cause of disability and ill health and has been linked to poor mental development and school achievement as well as behavioural abnormalities (Martins *et al.*, 2011). Through its effects on health, undernutrition increases healthcare costs, reduces productivity and slows economic growth (WHO, 2017).

In low- and middle-income countries (LMICs), more than a quarter of children under five (∼148 million) suffered from stunting in 2018. In the same year, ∼48 million suffered from wasting, with nearly 16 million cases of severe wasting (UNICEF, 2019; UNICEF *et al.*, 2019). The economic costs of undernutrition, in terms of lost national productivity and economic growth, are equivalent to ∼11% of GDP in Africa and Asia each year (World Bank, 2019a). Undernutrition is therefore an important public health problem and remains a major challenge for the majority of the LMICs.

Evidence shows that timely intervention can prevent adverse effects of undernutrition (Bhutta *et al.*, 2008; Menon *et al.*, 2018). However, due to limited resources, it is not possible to fund every effective intervention, and it is necessary to take decisions regarding which course(s) of action to follow. For this reason, economic evaluation (i.e. the comparative analysis of alternative courses of action in terms of both their costs and consequences) becomes necessary (Drummond *et al.*, 2015).

A recent overview of systematic reviews of effectiveness (Salam *et al.*, 2015) categorized nutrition interventions into four umbrella areas: preventive; therapeutic; fortification strategies; and delivery platforms (Table 1). According to World Health Organization guidelines, these various approaches should be regarded as complementary, with their relative importance depending on local conditions and the specific mix of local needs. For example, programmes that deliver micronutrient supplements often provide the fastest improvement in the micronutrient status of individuals or targeted population groups. Food fortification tends to have a less immediate but nevertheless a much wider and more sustained impact (Allen *et al.*, 2006). To assist in the prioritization of the most valuable set of interventions, economic evaluations should ideally take a consistent approach to the characterization of costs, effects and value for money.

**Table 1:** Categories adapted from Salam *et al.* (2015)

Topic area	Description	Examples
Preventive nutrition interventions	Interventions to prevent undernutrition and micronutrient deficiencies	Preventive zinc supplementation; breast feeding; complementary feeding; preventive multiple micronutrient supplementation
Therapeutic nutrition interventions	Interventions to treat undernutrition and micronutrient deficiencies	Ready-to-use therapeutic food for community management of severe acute malnutrition; therapeutic zinc supplementation; therapeutic multiple micronutrient supplementation
Fortification strategies	Deliberately increasing the content of an essential micronutrient in food to improve the nutritional quality of the food supply	Single nutrient fortifications
Delivery platforms	Specific modes and channels of delivering interventions	Conditional cash transfers

The aim of this systematic review is to provide an overview of economic evaluations of nutrition-specific interventions aimed at tackling undernutrition in LMICs. We focus on a specific set of recommended nutrition-specific interventions and programmes (Black *et al.*, 2013; Bhutta *et al.*, 2013; 2008) and compare and contrast evaluations of interventions in different areas.

## Methods

### Search methods for identification of studies

In 2015, Disease Control Priorities (DCP) 3rd edition (Black *et al.*, 2016) published a review of cost-effectiveness studies on global reproductive, maternal, newborn and child health that included nutrition interventions. In our review, we included all nutrition studies reported in DCP, and the Tufts Global Health Cost-Effectiveness Analysis database (Tufts Center for the Evaluation of Value and Risk in Health). We adapted the DCP search strategy to identify studies included in MEDLINE and Embase database from 2015 to March 2019. Finally, we reviewed the reference lists of any systematic reviews we identified for additional studies. Search strategies are reported in the Supplementary Material.

### Study eligibility

The focus was restricted to full economic evaluations of nutrition-specific interventions in low- and middle-income countries (World Bank, 2019b). We defined nutrition-specific interventions and programmes, as in Black *et al.* (2013). We only included economic evaluations that directly looked at nutrition-related outcomes (Bhutta *et al.*, 2008; 2013). We excluded studies on other forms of malnutrition (such as obesity and excess calorie intake) and economic evaluations without a specific focus on the health impacts of inadequate micronutrient or energy intake (e.g. cost-effectiveness analyses of producing and delivering fortified food, or evaluations assessing impacts on cognitive development outcomes only).

### Data extraction and categorization

We assessed the quality of the eligible studies using the Consolidated Health Economic Evaluation Reporting Standards guideline (Husereau *et al.*, 2013). For each study, if applicable, we rated each item as ‘satisfied’, ‘partially satisfied’ or ‘not satisfied’. For each study, we extracted information on the setting, target population, type of intervention and comparator, type of economic evaluation, method for the estimation of the treatment effect, type of health and non-health outcomes included, categories of costs considered and presence and type of analytical model and summary results. A data extraction form is included in the Supplementary Material. All articles chosen for extraction were read by two reviewers and any discrepancies were resolved by discussion with a third.

In this review, a distinction was drawn between impacts on private consumption (e.g. out-of-pocket expenditures) and productivity. Productivity refers to the value of current and future goods and services produced by an individual and includes both formal paid production and informal production (e.g. domestic labour and caring responsibilities). We included consumption incurred by individuals (i.e. due to purchases of goods in private markets) in the costs, whereas impacts on productivity and ability to work (i.e. value of goods and services produced by an individual) were considered as non-health outcomes of the intervention.

We distinguished costs falling within and outside the health system. The first group included costs incurred by the health system and public expenditure in the health sector. With costs falling outside the health system, we distinguished those eligible for public funding in other public sectors (e.g. agriculture, education), private sector expenditure (e.g. costs of fortification programmes incurred by food manufacturers) and out-of-pocket expenditure and household costs. We did not make any currency adjustment.

Some authors did not explicitly report the use of a decision model; however, in their analysis, the risk of experiencing different events was combined with specific costs and outcomes to obtain results. For example, a given level of a nutrition deficiency (vitamin A) was linked to a probability of ill health events (e.g. Bitot’s spots, night blindness and blindness) to which were attached health-related quality of life scores and costs. In other words, the economic evaluation was implicitly based on a decision tree model with a probability node leading to specific events, to whose specific costs and outcomes were attached. We classified such analyses as economic evaluations based on a decision tree model.

We categorized studies according to: types of intervention, types of economic evaluation and perspectives on costs and outcomes. Where a study included multiple economic evaluations of different interventions or strategies belonging to multiple topic areas, the evaluation was included in all relevant categories. To better illustrate the challenge of comparing across studies, we developed a case study based on strategies to combat zinc deficiency in China.

## Results

### Search results

**Figure 1:**
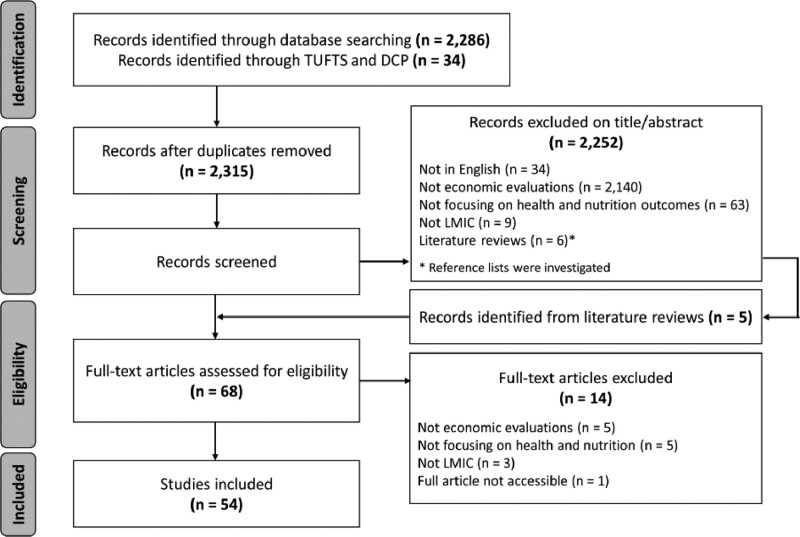
PRISMA flow diagram

Data on numbers of records, abstracts read and articles that qualified for data extraction are given in the flowchart in Figure 1.

The 54 studies identified by our review provided 62 separate economic evaluations across 4 topic areas (4 studies reported economic evaluations of interventions belonging to 2 different topic areas each; 2 studies reported economic evaluations of interventions belonging to 3 different topic areas). Detailed information about setting, target population, type of intervention and comparator, type of evaluation and main results of included economic evaluations is found in Supplementary Table S1. Supplementary Table S2 summarizes the main characteristics of identified economic evaluations: method for the estimation of the treatment effect; type of health and non-health outcomes included; categories of costs considered; and presence and type of analytical model.

### Types of intervention

**Figure 2:**
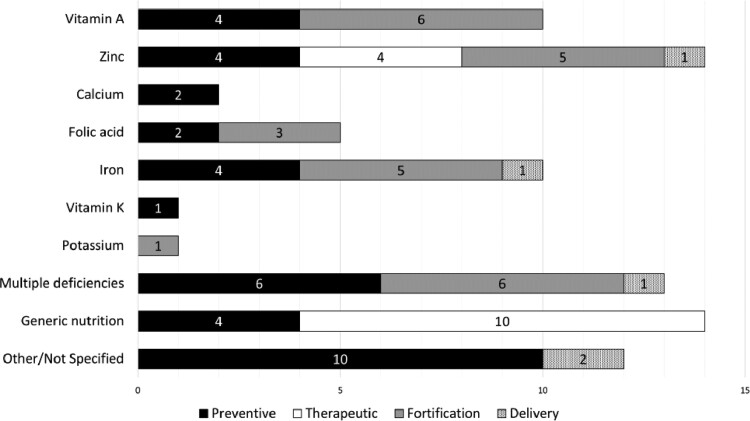
Deficiencies addressed. Total number of economic evaluations (82) is higher than the total number of studies (54) because some studies included interventions that address various deficiencies and were therefore included in all relevant categories

Using the categories reported in Table 1, we characterized the interventions accordingly: 22 (36%) fortification strategies; 23 (37%) preventive; 12 (19%) therapeutic; and 5 (8%) delivery platforms. The fortification strategies comprised mainly (77%) fortification of rice, oil, sugar or wheat to address specific nutritional deficiencies, such as vitamin A, zinc, iron or folic acid deficiency; the reminder addressed multiple deficiencies. Preventative interventions addressed mainly (74%) specific nutritional deficiencies (i.e. vitamin A, zinc, iron, calcium, energy/protein, vitamin K and folic acid). Three out of four economic evaluations of therapeutic nutrition interventions analysed community-based approaches to address generic malnutrition and provide supplementary nutrition. Delivery platforms included, e.g. price subsidies and franchising interventions to address iron, zinc and other multiple deficiencies (Figure 2).

### Types of economic evaluation

**Figure 3:**
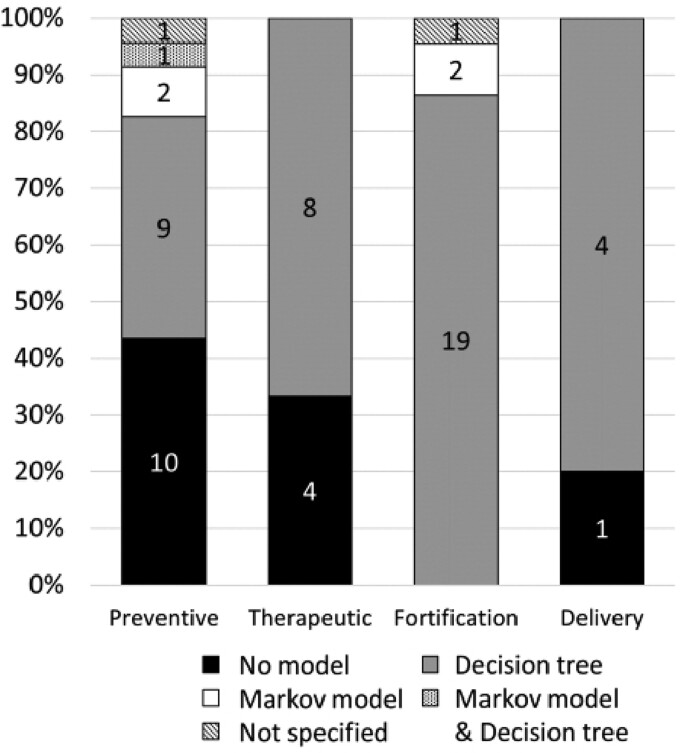
Type of decision model by topic area. Total number of economic evaluations (62) is higher than the total number of studies (54) because some studies conducted economic evaluations of multiple interventions belonging to different areas

We identified 56 (90%) CEAs, 1 (2%) CEA and cost–benefit analysis (CBA) conducted in parallel, 4 (7%) CBAs and 1 (2%) social return on investment (SROI). All CBAs and SROIs evaluated preventive interventions. Across all topic areas, 45 (73%) economic evaluations included a decision model; 40 (65%) were based on a decision tree and 4 (6%) on a Markov model; 1 analysis was based on a combination of decision tree and Markov model; 2 evaluations did not report enough information to assess whether a model was used (Figure 3). Less than half (43%) of the economic evaluations of preventive interventions did not include a model and more than one-third (35%) were within-trial economic analyses (see Supplementary Table S2 for further details). By contrast, all evaluations of fortification strategies were based on a model, and the treatment effect estimates used in the economic evaluations of fortification strategies were usually (91%) based on previous literature or assumptions.

When a model was employed for the evaluation of preventive, therapeutic and delivery interventions, this was typically original (i.e. newly developed). Across these three topic areas, 24 evaluations included a model, of which 16 (63%) were original model, with the remainder using previous examples or tools (see Supplementary Table S2 for further details). By contrast, all evaluations of fortification strategies were based on a model, and 17 out of 21 (81%) were based on a previously developed model. Of note, 15 evaluations were based on various versions of the Harvest-Plus approach, which is designed to measure costs and health benefits of biofortified staple crops (Zimmermann and Qaim, 2004; Stein *et al.*, 2005).

### Quality assessment

On average, studies reported that 85% of the items included in the checklist. However, in 31 studies (57%), the perspective was not explicitly specified and its impact on costs was not discussed. Of note, the perspective item was reported in only 22% and 23% of the economic evaluations of preventive and fortification strategies, respectively. By contrast, the same element was reported in 75% and 80% of the economic evaluations of therapeutic interventions and delivery platform, respectively. Furthermore, out of 39 studies based on a model, 12 (31%) did not explain the choice of model and 10 (26%) did not describe and give adequate reasons for the specific type of decision analytical model used. Of note, model choice item was not satisfied in 42% and 32% of the economic evaluations of preventive and fortification strategies, respectively. By contrast, all economic evaluations of delivery platform and 78% of therapeutic interventions reported the model choice item. The complete report of the quality assessment is reported in Supplementary Table S3.

### Perspectives on costs and outcomes

We grouped the studies according to the components included in the primary result of the economic evaluation (i.e. type of outcome and costs categories that compose the incremental cost per outcome for CEAs; costs and benefits included in the cost–benefit ratio and return on investment for CBAs and SROIs, respectively). Overall, studies measured a great variety of health outcomes including mortality, years of life lost, generic health outcomes (such as DALYs and QALYs), nutrition-related disease-specific outcomes (such as anaemia, Bitot’s spots, night blindness, corneal scarring, blindness, diarrhoea, neural tube defects), monetary values and disease-specific outcomes not directly linked to undernutrition (e.g. malaria, HIV infections).

**Figure 4:**
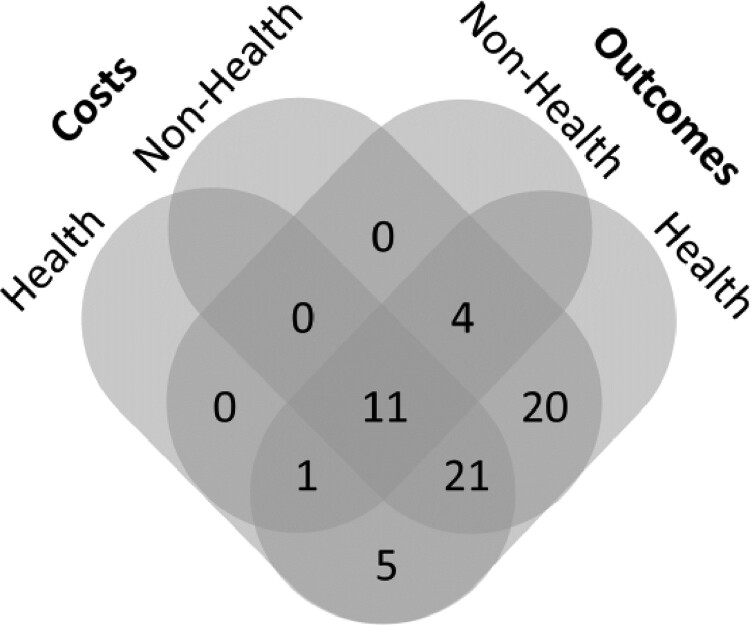
Health and non-health costs and outcomes

Six economic evaluations (10%) considered only costs falling within the health system. These were all analyses of preventive and therapeutic interventions. Of these, 5 (83%) considered only health outcomes (i.e. DALYs and/or other specific health outcomes); the remaining study included also non-health outcomes (productivity and education). Twenty-four economic evaluations (39%) considered only costs falling outside the health system. Of these, 20 (83%) reported health outcomes only; the 4 (17%) remaining analyses included also non-health outcomes. Thirty-two economic evaluations (52%) included costs falling both within and outside the health system. Of these, 21 (66%) reported only health outcomes; the 11 (34%) remaining analyses reported both health and non-health outcomes. Figure 4 shows that numerous studies adopted different perspectives for the evaluation of costs and outcomes. Of note, almost all analyses that measured only health outcomes but considered exclusively costs falling outside the health system and ignored costs (savings) for the health system were economic evaluations of fortification strategies.

Out of the 27 evaluations of fortification and delivery strategies, 26 (96%) reported main results using a generic measure of health (25 reported DALYs, 1 reported QALYs). By contrast, 16 (46%) out of 35 evaluations of preventive and therapeutic interventions reported results only in terms of impacts on specific health outcomes. Overall, across the four topic areas, 16 (26%) evaluations included non-health outcomes. Of these, 11 reported productivity outcomes (i.e. impacts on income, wages, days of work, and vegetable production) and 5 reported both productivity and education outcomes (i.e. cognitive development, school performance, and years of education) (see Supplementary Table S4 for further details).

To illustrate the challenge in interpreting studies with different perspectives, in Box 1, we present a scenario describing the interpretation of cost-effectiveness evidence to inform the development of a national strategy to combat zinc deficiency.

Box 1 Zinc case study

***Alternative strategies to address zinc deficiency in China***

In this example, we consider the use of published economic evidence to inform investment in addressing zinc deficiency in China. Our review of the evidence (see Supplementary Table S5) identified a set of studies including the following evidence-based policy options:

- Preventative supplementation (Edejer *et al.*, 2005; Ma *et al.*, 2008; Bhutta *et al.*, 2013; Fink and Heitner, 2014);
- Fortification of wheat (Wang *et al.*, 2016), rice (Zhang *et al.*, 2018), or combinations of different staple foods (Edejer *et al.*, 2005; Stein *et al.*, 2007; Ma *et al.*, 2008);
- Delivery platform based on social franchising (Bishai *et al.*, 2015); and
- Therapeutic supplementation (Robberstad *et al.*, 2004; Mejia *et al.*, 2015; Shekar *et al.*, 2016; Shillcutt *et al.*, 2017).

For the sake of simplicity, we compare all strategies as independent and do not consider combinations not assessed within the supporting studies. Cost-effectiveness estimates are summarized in Supplementary Table S6. We did not adjust the currencies to the same year, but we reported in the table the currency year and study year for each study.

*Setting and location*

The studies by Ma *et al.* (2008), Zhang *et al.* (2018) and Wang *et al.* (2016) were set in China. The study by Edejer *et al.* (2005) was set in South-East Asia and sub-Saharan Africa. Fink *et al.* (2014) considered a representative low-income country. Bhutta *et al.* (2013) considered 34 countries, but not China. The study by Bishai *et al.* (2015) was set in Myanmar; Mejia *et al.* (2015) in Colombia; and Robberstad *et al.* (2004) in the United Republic of Tanzania. The studies by Shillcutt *et al.* (2017) and Stein *et al.* (2007) were set in India.

*Preventive and fortification strategies*

Ma *et al.* (2008) indicated that cost per DALY of fortification compared to doing nothing was lower than cost per DALY of supplementation compared to doing nothing. However, comparators were not clearly specified and it was not clear whether results were expressed as average or incremental cost effectiveness ratios (ICERs) compared to a scenario of doing nothing. Furthermore, costs falling on the health system were not considered. By contrast, Edejer *et al.* (2005) did include healthcare costs and found that supplementation was more costly, but more effective than fortification, when compared with a scenario of doing nothing. However, ICERs of supplementation compared to fortification were not reported^1^.

Wang *et al.* (2016) and Zhang *et al.* (2018) did not consider costs falling on the health system and reported incremental costs of $226–594 and $311–4989 per DALY averted for fortification strategies vs doing nothing, respectively. These ICERs are much higher compared to those reported in Edejer *et al.* (2005) ($14 and $55 per DALY averted in South East Asia and Sub-Saharan Africa, respectively). Stein *et al.* (2007) adopted the same approach as Wang *et al.* (2016) and Zhang *et al.* (2018) and found more favourable ICER of $0.73–7.31 per DALY averted for fortification vs doing nothing in India.

Fink *et al.* (2014) estimated an incremental cost of $606–1211 per DALY averted for preventive supplementation interventions vs doing nothing. However, it was not clear whether health system costs were also included or not. Bhutta *et al.* (2013) argued that scaling up preventive supplementation was cost effective in various countries but did not consider China.

*Therapeutic interventions and delivery platforms*

Bishai *et al.* (2015) estimated an incremental cost of $214 (societal perspective) or $339 (medical perspective) per DALY averted for a delivery platform based on social franchising vs doing nothing. Both sets of results included healthcare costs, and the flexibility in the perspectives considered for the evaluation could facilitate comparison across studies. However, according to the authors, results may not be generalizable outside Myanmar because the organization promoting the programme was fortunate to have a large provider network and donor support.

Mejia *et al.* (2015) found therapeutic zinc supplementation to be dominant (i.e. less costly and more effective in reducing chances of diarrhoeal episodes and death) compared to standard treatment from the Colombian health system perspective. Shillcutt *et al.* (2017) compared the same strategy to do nothing in India from a societal perspective. However, because results were expressed in terms of cost per case averted, a comparative value of DALY to disease case would be required to perform a comparison with alternative strategies. Robberstad *et al.* (2004) estimated an ICER of $40 per DALY averted for therapeutic supplementation compared to standard treatment in the United Republic of Tanzania. However, the evaluation included both health system and non-health system costs. Shekar *et al.* (2016) estimated health outcomes, healthcare and non-healthcare costs of scaling up therapeutic zinc supplementation and found that was cost effective in various countries but did not consider China.

*Summary*

Caution is needed when using ICERs from reported studies, to avoid comparing results considering different cost categories. Studies of interventions provided in different sectors often consider different costs and outcomes, with relevance determined by the audience for the analysis. Similar studies in different settings give different ICERs, and ordering of fortification and supplementation does not appear to be consistent across studies. Preventative strategy may have the highest ICER, but it is unclear if this includes healthcare costs, which could lower the ICER. Therapeutic and delivery strategies may have the lowest ICERs; however, there is no economic evidence for such strategies in China. In conclusion, studies are difficult to compare, and it is challenging to use the available evidence to inform the identification of the optimal strategy to be adopted.

## Discussion

### Main findings

We found that CEA was the most common analytical technique employed for the economic evaluation of interventions that address undernutrition across all settings. We observed differences in CEAs of interventions delivered within the health sector (e.g. preventative and therapeutic nutrition interventions) and outside the health sector (e.g. fortification strategies and other delivery platforms such as price subsidies and conditional cash transfers). However, while we observed clear differences in the categories of costs and outcomes included, we cannot say whether this is due to the studies aiming to inform different decision-makers. Most studies did not make clear whether they aimed to inform decisions within the public sector, and authors frequently failed to report the perspective adopted for the evaluation or who pays for the intervention.

The choice of costs and outcomes considered in the studies was also influenced by the degree of use of evaluation tools, such as the widespread use of the Harvest-Plus approach (Stein *et al.*, 2005) as reference model to evaluate fortification strategies. We acknowledge that the tool was developed with the aim of providing a common framework for CEA of biofortification (Stein *et al.*, 2007; 2008). However, the reporting quality assessment in this review highlighted that the tool was not accompanied by any reflection from authors on appropriateness of perspective, model structure and outcomes and costs included. By contrast, in studies where newly developed models were employed, the appropriateness and justification of the perspective adopted were discussed, reflecting on which costs to include in the model (Robberstad *et al.*, 2004; Desmond *et al.*, 2008; Wilford *et al.*, 2012; Chola *et al.*, 2015).

In principle, the costs and outcomes in sectors outside the intervention funder should not be ignored or that would risk prioritizing interventions that impose costs on others, or conversely undervaluing interventions. An impact on health is one of the core objectives of nutrition interventions, and so it would seem relevant to consider health outcomes. Impacts on healthcare resource use produce opportunity costs in terms of health production through alternative uses of those resources. The boundaries for cost inclusion therefore should align with the boundaries for outcome inclusion, driven by examination of the opportunity costs of the resources impacted by the intervention. Outcomes and costs categories should reflect all the relevant impacts of the intervention, and should be capable of adequately inform the decision-makers. For example, economic evaluations of zinc fortification interventions should consider costs included in the health sector, particularly if interventions benefits are measured using health outcomes.

Evaluations of preventive and therapeutic interventions frequently reported result only in terms of impacts on disease-specific health outcomes, which might be less useful to inform comparisons across topic areas. Most of the analyses of preventive and therapeutic interventions that reported generic outcome measures (e.g. DALYs) included costs outside the health sector without considering the outcomes that may be relevant outside the health sector, e.g. impacts on productivity and earnings. Variations in DALYs averted were frequently compared to costs falling within and outside the health system accrued together. By contrast, most evaluations of fortification strategies measured impacts of the intervention using DALYs but considered costs falling outside the health system only. Because evaluations of preventive and therapeutic interventions typically included also costs falling within the health system, resulting costs per DALY will be systematically different from those of fortification strategies, and not directly comparable.

Evaluations of preventative and therapeutic nutrition interventions sometime included impacts on non-health outcomes such as productivity and education. In contrast, evaluations of fortification and delivery strategies frequently focused on impacts on health only, and ignored non-health effects. Fortification and delivery strategies might thus systematically omit relevant impacts of the intervention.

Studies that adopted a broader perspective frequently aggregated all incremental costs and compared them to an incremental outcome estimate. However, adding up monetary costs that fall on different budgets may not adequately capture the opportunity costs (i.e. what is forgone in order to accommodate the resources to provide new services) (Sculpher *et al.*, 2017). Given the different remits and forgone activities, an additional dollar commanded from the health sector displaces a different set of activities and outcomes to a dollar commanded from the agricultural sector. By accruing all costs together, evaluations of preventative and therapeutic nutrition frequently fail to distinguish impacts that fall on different sectors and under the remit of different decision-makers (Walker *et al.*, 2019). Health and non-health effects and costs falling within and outside the health system should be instead identified separately (Sculpher *et al.*, 2014).

### Strengths and limitations

We did not include nutrition-sensitive interventions and so our review is not inclusive of the full range of multisectoral nutrition-sensitive approaches that has been recommended by Scaling Up Nutrition and other nutrition advocacy groups [United Nations Scaling Up Nutrition (SUN)/REACH Secretariat, 2016]. However, even with the narrower focus on nutrition-specific interventions and outcomes, we identified heterogeneity in the approach taken to economic evaluation. Our review is not exhaustive, but identifying more studies would be unlikely to alter this picture of heterogeneity in costs and outcomes.

A similar systematic review by Gyles *et al.* in 2012 provided an overview and summary of studies of nutrition interventions in which health-related economic implications of the intervention have been addressed, and considered a wider spectrum of strategies. Our study focuses specifically on the context of nutrition interventions delivered in LMICs, considers the methodological aspects of each study and provides an overview of more recent literature. However, findings were similar, as the authors found that approaches and methodologies to calculate health-economic impacts of nutrition interventions were sometimes *ad hoc* in nature and vary widely in quality (Gyles *et al.*, 2012).

Other economic evaluations of undernutrition have previously been included but not distinguished, in broader systematic reviews that focused on reproductive, maternal, newborn and child health (Black *et al.*, 2016) and on generic health interventions designed to mitigate disease burden (Neumann *et al.*, 2016). Other previous systematic reviews focused on the economic evaluation of specific intervention strategies to address undernutrition, such as community health worker interventions (Nkonki *et al.*, 2017), food fortification (Detzel and Wieser, 2015), and early childhood nutrition and development interventions (Batura *et al.*, 2015; Hurley *et al.*, 2016). However, previous reviews found results consistent with ours. Similar issues were highlighted, such as: differences in methods for standardizing costs (Black *et al.*, 2016), lack of standardization in effectiveness measures across nutrition interventions conducted in different domains (Salam *et al.*, 2015) and consequent difficulties to compare the cost-effectiveness of interventions due to differences in outcome measures (Batura *et al.*, 2015). A move towards a common outcome measure (e.g. cost-per-DALY averted) was advocated to facilitate the comparison of cost-effectiveness between studies and contexts (Batura *et al.*, 2015). Of note, on the one hand, the extent to which the cost-per-DALY literature has addressed nutritional deficiencies is much lower compared to other high burden disease areas in LMICs (e.g. HIV/AIDS, tuberculosis, neglected tropical diseases and malaria) (Neumann *et al.*, 2016). On the other hand, a general scarcity of studies on early childhood nutrition and development interventions conducted from societal perspective was pointed out (Batura *et al.*, 2015), together with the need to consider broader benefits than life saved specifically in the evaluation of nutrition interventions (Black *et al.*, 2016).

A standardized approach for the economic evaluation of nutrition interventions may be difficult to establish due to the differences in the methods traditionally adopted in the various sectors. Methods for economic evaluation within the health sector have diverged from methods typically employed outside the health sector, and analyses are unlikely to be equivalent and may yield different resource allocation decisions (Bala *et al.*, 2002). However, better transparency about perspective adopted, outcomes and costs considered and assumptions employed would facilitate the comparison across studies from different areas, even if based on different approaches.

We acknowledge the difficulties and limitations of using checklists to evaluate the quality of such a heterogenous set of studies (Wijnen *et al.*, 2016). Economic evaluations involved strategies that may be enacted by different decision-makers from different settings and therefore might need to accommodate needs and preferences of different agencies. However, we showed that systematic differences and trends by type of intervention are not connected to countries and settings. Our research strategy included only studies published in peer-reviewed journals, in English. Grey literature was not investigated.

## Conclusion

Alternative strategies are available to address the same nutrition deficiencies. Comparing interventions across various topic areas becomes thus fundamental to assist decision-makers (such as the Ministry of Health or the Ministry of Agriculture) in prioritizing alternative nutrition interventions. In this review, we highlighted trends and systematic differences in the approach to the economic evaluation of interventions and strategies implemented within and across different sectors of the economy. Furthermore, we illustrated how inconsistencies in the approaches can undermine the ability to compare across studies and have important consequences for decision-making. We showed how the use of ‘off-the-shelf’ models and tools can conceal what outcomes, costs and value judgements are adopted in the economic evaluation.

We echo the recommendation by Gyles *et al.* (2012) who called for the development of an encompassing economic framework that would allow for a uniform and complete measurement of the economic costs and benefits borne by all stakeholders. For example, comparison across studies would be facilitated by the compilation of impact inventories that distinguish impacts on the different sectors and dimensions, as recommended by the Second panel on CEA (Sanders *et al.*, 2016; Walker *et al.*, 2019). Furthermore, following reporting standards as indicated in various reference cases (Wilkinson *et al.*, 2016; Vassall *et al.*, 2017; Robinson *et al.*, 2019) would help to promote consistency and improve the quality of future studies, thus facilitating their comparability and decision-making.

## Supplementary Material

czaa149_Supp

## Endnotes

1 This is because the study compared various strategies and combinations of treatments addressing also deficiencies other than zinc, and these specific zinc interventions were dominated by other alternative interventions. However, with available data, ICERs could be calculated.

## Precis

A systematic review and critique of economic evaluations of interventions to address undernutrition in low- and middle-income countries delivered within and outside the health sector.

*Ethical approval.* No ethical approval was required for this study.
