# Supplementary material for: Economic evaluation of interventions to address undernutrition: a systematic review
Source: Health Policy Plan. 2020 Dec 6;36(4):533–41. doi: 10.1093/heapol/czaa149 (PMC8128006; doi:10.1093/heapol/czaa149)
Supplement: czaa149_Supp [file czaa149_supp.zip › Appendix_final.docx]

# Supplementary Material

## Table S1: Summary of the included studies

| **1. Preventive nutrition interventions** | | | | | | |
| --- | --- | --- | --- | --- | --- | --- |
| **Authors (year)** | **Country** | **Target population** | **Intervention** | **Comparator** | **Type of evaluation** | **Main results** |
| Awasthi et al. (2000) | India | Pre-school children aged between 1.5 and 3.5 years | Improve the nutritional status of pre-school children through periodical deworming | Placebo | CEA | R.543 per case of stunting prevented with albendazole over placebo |
| Alonzo Gonzalez et al. (2000) | Tanzania | Infants under-1-year-olds | Iron supplementation | Standard case management | CEA | $9.7-$10.2 per DALY averted |
| Baltussen et al. (2004) | African, South American, European, and Southeast Asian sub-regions | Pregnant women during antenatal visits | Iron supplementation | No iron-deficiency control program | CEA | All interventions in the 4 regions have ICER lower than three times GDP per capita |
| Bernal and Fernandez (2013) | Colombia | Low-income children under the age of 6 | Home-based childcare program including supplemental nutrition and psychosocial stimulation | No intervention | CBA | Benefit-cost ratio ranged from 1.0 to 2.7, depending upon varying discount rates. |
| Bhutta et al. (2013) | 34 countries | Adolescents, women of reproductive age, pregnant women, newborn babies, infants, and children | Periconceptional folic acid supplementation; maternal balanced energy protein supplementation; maternal calcium supplementation; multiple micro nutrient supplementation in pregnancy; promotion of breast feeding; appropriate complementary feeding; vitamin A and preventive zinc supplementation in children 6–59 months of age | Present level of coverage of the intervention | CEA | Interventions are well within the cost-effectiveness benchmark (less than three-times per person income) for all countries |
| Chola et al. (2015) | Uganda | Mothers and their 0 to 6 month old children | Peer Counselling for the Promotion of Exclusive Breastfeeding | Standard health facility breastfeeding promotion services (HFP) | CEA | ICERs: US$68 per month of exclusive or predominant breastfeeding; U$11,353 per DALY averted |
| Chow et al. (2010) | India | Preschool children and new and expectant mothers | High-dose vitamin A supplementation | No intervention | CEA | ICER: US$23–$50 per DALY averted |
| Danielsson et al. (2004) | Vietnam | Infants aged 1–13 weeks | Routine vitamin K prophylaxis | No intervention | CEA | US$87 (£48, J72) per DALY saved |
| Desmond et al. (2008) | South Africa | HIV infected mothers | Exclusive Breastfeeding (EBF) Support Programme | No intervention | CEA | ICERs (per additional MEBF): Nothing – Basic R616 ($88); Basic – Simplified R162 ($23); Simplified – Full R879 ($126) |
| Edejer et al. (2005) | Developing countries | Children under 5 | Supplementation with vitamin A or zinc; provision of supplementary food with counselling on nutrition | Do nothing scenario | CEA | Provision of supplementary food and counselling on nutrition is the least cost effective (compared to alternatives such as fortification with zinc or vitamin A) |
| Feldhaus et al. (2016) | Nepal | Pregnant women | Calcium supplementation in addition to the existing standard of care | Standard of care; No treatment | CEA | $25.33 ($25.22–29.50) per DALY averted compared to MgSO4 treatment |
| Fink and Heitner (2014) | Representative low income country | Children of ages 1–5 | Three delivery mechanisms: the direct distribution of zinc supplements, the distribution of micronutrient biscuits including zinc, and the distribution of zinc through water filtration systems | No intervention | CEA | Estimated cost per DALY is US$ 606 for pill supplementation, US$ 1211 for micronutrient biscuits, and US$ 879 per DALY saved for water filtration systems |
| Goudet et al. (2018a) | Kenya | Pregnant and breastfeeding women and mothers of young children | Home-based counselling by community health volunteers (CHVs) for the promotion of optimal maternal infant and young child nutrition (MIYCN) practices. The intervention aimed to improve breastfeeding and other infant feeding practices | No intervention | SROI | $USD 1 invested was estimated to bring USD$ 71 (sensitivity analysis: USD$ 34–136) of social value for the stakeholders. |
| Lopez Boo et al. (2014) | Nicaragua | Children between six and sixty months of age | Micronutrient supplementation (Sprinkes) and early childhood stimulation program in the context of PAININ (Programa de Atencion Integral a la Ninez Nicaraguense), an integrated nutritional and early childhood development (ECD) program | No intervention | CBA | Cost–benefit ratio of 1.50 from the PAININ plus sprinkles package |
| Ma et al. (2008) | China | Whole population | Iron and zinc supplementation;  Dietary diversification through health education | No intervention | CEA | Supplementation: I$179 (iron), I$399 (zinc); dietary diversification: I$103 (iron), I$103 (zinc) |
| Qureshy et al. (2013) | Indonesia | 9-month to 2-year children | Enhanced service package (Generasi) that includes height measurement, intensified personal counselling on nutrition, hygiene, active feeding and prevention and control of diarrhoea, including counselling on the use of zinc as adjunct therapy in its treatment, accompanied with improved training and financial incentives for cadres, provision of multi-micronutrient sachets and a sanitation triggering campaign | Existing Posyandu services | CBA | Benefit–cost ratio: 2.08 |
| Schreinemachers et al. (2016) | Bangladesh | Women | Training in home gardening and nutrition | No intervention | CEA | US $3,059 per DALY saved |
| Shaheen et al. (2015) | Bangladesh | Pregnant women | Invitation to food supplementation early in pregnancy combined with multiple micronutrient supplements (MMS) | Invitation to food supplementation at usual time in pregnancy combined with iron-folic acid | CEA | One extra life year saved at US$30, US$27, and US$34, for NGO run CNC, government run CNCs, and a hypothetical scenario of highest cost, respectively. |
| Sharieff et al. (2006) | Pakistan | Two fictitious communities representative of the Pakistani population | Home-fortification program using zinc, iron and iodine, vitamins C, D and A, and folic acid (via ‘Sprinkles’ single-dose sachets) | No intervention | CEA; CBA | $12.2 ($8-$97) per DALY saved. Present value (i.e. benefit:cost return) of the gain in earnings is $37 ($18- $51) for each dollar spent |
| Shekar et al. (2016) | Democratic Republic of the Congo (DRC), Mali, Nigeria and Togo | General population, mothers of children 0–59 months of age, children 6–59 months of age and pregnant women | Community nutrition programmes for growth promotion; Multiple micronutrient powders; Iron–folic acid supplementation; Vitamin A supplementation; Complementary food for the prevention of moderate acute malnutrition | Present level of coverage of the intervention | CEA | Considered individually, all interventions except provision of complementary food for the prevention of moderate acute malnutrition are ‘very cost-effective’. |
| Svefors et al. (2018) | Bangladesh | Pregnant women | Early food and multiple micronutrients (MMS) supplementation | Usual timing of food and routine iron and folate supplementation | CEA | ICER per 5 years and lifetime DALY averted: US$187 and US$24, respectively |
| Walters et al. (2016) | Vietnam | Pregnant and breastfeeding women and mothers of children <2 year old | National breastfeeding promotion strategy | No intervention | CBA | Intervention can prevent 200 child deaths per year and generate monetary benefits of US$2.39 for every US$1, or a 139% return on investment |
| Waters et al. (2006) | Peru | Children less than 2 years of age | Infant nutrition education programme | No intervention | CEA | Intervention costs US$6.12 per child reached, US$55.16 per case of stunting prevented, US$1952 per death averted |

| **2. Therapeutic nutrition interventions** | | | | | | |
| --- | --- | --- | --- | --- | --- | --- |
| **Authors (year)** | **Country** | **Target population** | **Intervention** | **Comparator** | **Type of evaluation** | **Main results** |
| Akram et al. (2016) | Pakistan | Severely malnourished children | Community-based therapeutic feeding programme (CTFP) to rehabilitate severely malnourished children using a high-density diet (HDD) made from indigenous ingredients (‘Baby Active’, also known as ‘sprinkles’) | No intervention | CEA | The net cost of rehabilitating a single child amounted to USD 34.31. The supplement was cost-effective in comparison to imported therapeutic foods |
| Bachmann (2009) | Zambia | Children under five with SAM | Community-based therapeutic care (CTC) for children with severe acute malnutrition | No intervention | CEA | CTC costs $ 53 (95% CI $18–$306) per DALY gained |
| Bhutta et al. (2013) | 34 countries | Adolescents, women of reproductive age, pregnant women, newborn babies, infants, and children | Management of SAM; management of MAM | Present level of coverage of the intervention | CEA | Interventions are well within the cost-effectiveness benchmark (less than three-times per person income) for all countries |
| Goudet et al. (2018b) | India | Pregnant women and children younger than 3 years | Adding a community based prevention and treatment for acute malnutrition intervention (Aahar) to standard care | Standard ICDS services alone | CEA | $23 per DALY averted (95%UI:19–28) |
| Mejia et al. (2015) | Colombia | Children younger than 5 years | Zinc supplementation for the treatment of acute diarrhea | Standard treatment without zinc | CEA | The zinc strategy is more effective and of a lower cost |
| Puett et al. (2013) | Bangladesh | Children 6–36 months of age | Community-based management of severe acute malnutrition (CMAM) | Standard care (i.e. inpatient treatment of SAM); no treatment | CEA | CMAM vs no treatment: US$26 per DALY averted Standard care vs no treatment: US$1344 per DALY averted |
| Robberstad et al. (2004) | United Republic of Tanzania | Children aged less than 5 years | Zinc as adjunct therapy to current standard treatment for children with non-dysenteric diarrhoea; Zinc as adjunct therapy to current standard treatment for all children with acute diarrhoea, including those with dysentery | Standard case management with oral rehydration salt (ORS) | CEA | The optimal treatment would be the provision of zinc in addition to ORS to all patients. However, confidence limits are so wide that extended dominance is not certain |
| Rogers et al. (2018) | Mali | Children aged 6–59 months | Treatment of uncomplicated severe acute malnutrition (SAM) by community health workers (CHW) | No intervention | CEA | Cost per child recovered: 259 USD (CHW-delivered care); 501 USD (outpatient facility-based care) |
| Rogers et al. (2019) | Pakistan | Children aged 6–59 months | Treatment for uncomplicated SAM delivered by Lady Health Workers (LHWs) employed within the national health system and complemented with non-governmental organisation (NGO) delivered outpatient facility-based care | No intervention | CEA | 146 USD per additional child recovered by outpatient facilities compared to LHWs |
| Shekar et al. (2016) | Democratic Republic of the Congo (DRC), Mali, Nigeria and Togo | General population, mothers of children 0–59 months of age, children 6–59 months of age and pregnant women | Therapeutic zinc supplementation with ORS; Community-based management of severe acute malnutrition (CMAM) | Present level of coverage of the intervention | CEA | Considered individually, all interventions are ‘very cost-effective’. |
| Shillcutt et al. (2017) | India | 2–59 month old children | Zinc and oral rehydration therapy for the treatment of acute child diarrhea | No intervention | CEA | Cost-effective with 95% certainty above $5.50 - $7.50 per appropriately treated child |
| Wilford et al. (2012) | Malawi | Children under 5 years of age | Existing health services with community-based management of acute malnutrition (CMAM) | Existing health services without CMAM | CEA | US$42-$493 per DALY averted |

| **3. Fortification strategies** | | | | | | |
| --- | --- | --- | --- | --- | --- | --- |
| **Authors (year)** | **Country** | **Target population** | **Intervention** | **Comparator** | **Type of evaluation** | **Main results** |
| Baltussen et al. (2004) | African, South American, European, and Southeast Asian sub-regions | Whole population (focus on effects on preschool children and new and expectant mothers) | Iron fortification | No intervention | CEA | All interventions in the 4 regions have ICER lower than three times GDP per capita |
| Bhutta et al. (2013) | 34 countries | Adolescents, women of reproductive age, pregnant women, newborn babies, infants, and children | Periconceptional folic acid fortification | Present level of coverage of the intervention | CEA | Interventions are well within the cost-effectiveness benchmark (less than three-times per person income) for all countries |
| Chow et al. (2010) | India | Whole population | Industrial fortification of mustard oil with vitamin A; biofortification with genetically modified mustard | Absence of an intervention | CEA | ICER: US$405–$450 per DALY |
| Dainelli et al. (2017) | China | Adult population | Milk powder fortified with potassium | Milk powder without potassium fortification | CEA | ICER: int$4711.56 per QALY in the best case scenario and assuming 100% compliance |
| De Steur et al. (2012b) | China | Whole population | Multi-biofortification of rice | Status quo without biofortification | CEA | US$2.3–$9.6 per DALY saved |
| De Steur et al. (2012a) | China | Whole population | Folate biofortified rice | No intervention | CEA | US$ 120.34 - US$ 40.1 per DALY saved |
| Edejer et al. (2005) | Developing countries | Children under 5 | Fortification of staple foods with vitamin A or zinc | Do nothing scenario | CEA | Fortification with zinc or vitamin A is the most cost effective intervention (compared to alternatives such as provision of supplementary food and counselling on nutrition) |
| Fiedler and Macdonald (2009) | 48 LMIC countries | Whole population | 122 food fortification (iron, vitamin A, and zinc) interventions | No intervention | CEA | The 60 most cost-effective interventions have costs per DALY saved ranging from US$1 to US$134 |
| Fiedler and Afidra (2010) | Uganda | Ugandan households and individuals (effects modelled on children < 5 yr, pregnant women, lactating women) | Fortifying vegetable oil and sugar with vitamin A | No intervention | CEA | Cost per DALY averted is US$82 for sugar and US$18 for oil. |
| Fiedler et al. (2013) | Zambia | Children < 5 years old, women of child bearing age | 14 food fortification (vitamin A, iron, zinc) portfolios | No intervention | CEA | The most cost-effective portfolio is vegetable oil, which has a cost per DALY saved ranging from 12% to 25% of that of sugar |
| Fiedler et al. (2015) | Bangladesh | Whole population | Industrial fortification (vitamin A, iron, zinc) interventions: wheat flour fortification; vegetable oil fortification; wheat flour and vegetable oil fortification | No intervention | CEA | Vegetable oil fortification is the most cost-effective of the three portfolios analysed (US$3.25 per DALY saved) |
| Fiedler et al. (2016) | Bangladesh | Children < 5 years old, pregnant and lactating women | Mola Promotion Program (MPP) aquaculture for Vitamin A supplementation | No intervention | CEA | $194 per DALY saved |
| Hoddinott (2018) | Zambia | Whole population (impacts modelled for: newborns, infants, and under-five children) | Folic acid fortification | No intervention | CEA | $14.90 per DALY averted |
| Lividini and Fiedler (2015) | Zambia | Entire population; children under 5 years of age;  women ages 15–49 years | Biofortification: high provitamin A maize | No intervention | CEA | $24 per DALY saved. Benefit-cost ratio (with fixed value of $1000 per DALY) of 42. Rate of return of 40% |
| Ma et al. (2008) | China | Whole population | Food fortification (iron and zinc) | No intervention | CEA | Iron: I$66 per DALY; zinc I$153 per DALY |
| Meenakshi et al. (2010) | 12 countries in Africa, Asia, and Latin America | Whole population | Biofortification of staple food crops with provitamin A, iron, and zinc | No intervention | CEA | Most costs per DALY averted for biofortification fall in the ‘‘highly” cost-effective category |
| Shekar et al. (2016) | Democratic Republic of the Congo (DRC), Mali, Nigeria and Togo | General population, mothers of children 0–59 months of age, children 6–59 months of age and pregnant women | Iron fortification of staple foods | Present level of coverage of the intervention | CEA | Considered individually, all interventions are ‘very cost-effective’. |
| Stein et al. (2006) | India | Children, pregnant and lactating women | Introduction of rice fortified with vitamin A (Golden Rice 2) | No intervention; Vitamin A supplementation | CEA | Even under pessimistic assumptions, the cost of saving one DALY is <$20 |
| Stein et al. (2007) | India | Infants (below 1 year of age) and children (1–5 years of age) | Zinc biofortification of rice and wheat | No intervention | CEA | $US 0.73–7.31 per DALY saved.  If DALY valued at $US 1000, IRRs range from 31% to 173% |
| Stein et al. (2008) | India | 4 target subgroups: children <= 5 years old, children aged 6-14 years, women >=15, men >=15 | Iron biofortification of crops (rice and wheat) | No intervention | CEA | Depending on the assumptions made, the cost per DALY saved ranges from 30 cents to US$8.70 |
| Wang et al. (2016) | China | Infants and children under 5 years of age | Biofortification of wheat with zinc | No intervention | CEA | US$ 226 – 594 per DALY saved |
| Zhang et al. (2018) | China | Whole population (impacts modelled for: Infants, Children under 5, Children 6-14, Men 15+, Women 15+, Pregnant women) | Agronomic Biofortification of Rice: foliar Fe or Zn spray alone; combination of both Fe and Zn spray with or without pesticides | No intervention | CEA | The cost of saving 1 DALYs ranged from US$376 to US$4989, US$194 to US$2730, and US$37.6 to US$530.1 for the single, dual, and triple foliar Fe, Zn, and/or pesticide application, respectively |

| **4. Delivery platforms** | | | | | | |
| --- | --- | --- | --- | --- | --- | --- |
| **Authors (year)** | **Country** | **Target population** | **Intervention** | **Comparator** | **Type of evaluation** | **Main results** |
| Bergmann et al. (2017) | Malawi and Mozambique | Children under 2 years of age | Various programmes for integrated HIV and nutrition service delivery | No intervention | CEA | Malawi: $11–29 per DALY averted; Mozambique: $16–59 per DALY averted |
| Bishai et al. (2015) | Myanmar | Children under 5 years | Public health intervention designed to promote uptake of ORS-Z in a community by adding ORS-Z as an additional product line in an existing social franchise program | Standard government and private sector practices | CEA | Median incremental cost per death averted: $5,955 (societal perspective); $8980 (medical perspective).  The median incremental cost per discounted DALY averted: $214 (societal perspective); $339 (medical perspective) |
| Plessow et al. (2016) | India | 6-23-Month-Old-Children | Price subsidies on fortified packaged infant cereals (F-PICs) to address iron deficiency anaemia (IDA) | No intervention | CEA | USD 909-3649 per DALY averted. In different scenarios, ICERs rage from -75 USD/DALY (cost-saving) to 150 USD/DALY  From a societal perspective, return per DALY averted (i.e. Net social cost / DALYs averted) ranges between gains of 1655 USD to a cost of 411 USD |
| Trenouth et al. (2018) | Pakistan | Poor households with children aged 6–48 months | Three cash-based interventions (CBIs): ‘double cash’ (DC) transfer; ‘standard cash’ (SC) transfer; ‘fresh food voucher’ (FFV) transfer | Standard care: interventions delivered by the underlying EU-WINS programme only | CEA | The cost per DALY averted: $845 - $1252 (using discounted and age-weighted methods), $434 - $641 (using non-discounted or age-weighted methods) |
| Wieser et al. (2018) | Pakistan | Households with 6–23-month-old children | Price subsidies on fortified packaged complementary foods (FPCF) | No intervention | CEA | The net cost per DALY of the interventions ranged from a return per DALY averted of $US 783 to $US 65 |

## Table S2: Main characteristics of the economic evaluations

| **1. Preventive nutrition interventions** | | | | | | | | | | | | |
| --- | --- | --- | --- | --- | --- | --- | --- | --- | --- | --- | --- | --- |
| **Authors** | **Method for the estimation of the treatment effect** | **Health Outcomes** | | | | | | **Non-health outcomes** | **Costs** | | **Model** | |
|  |  | **Mortality** | **YLL** | **Generic** | **Disease specific** | **Monetary values** | **Other** |  | **HS** | **Non-HS** | **Type** | **Reference** |
| Awasthi et al. (2000) | RCT | X | - | - | X | - | - | - | X | - | - | - |
| Alonzo Gonzalez et al. (2000) | RCT | X | X | DALY | X | - | Malaria | - | X | X | - | - |
| Baltussen et al. (2004) | Previously published studies | X | - | DALY | - | - | - | - | X | X | MM | Original |
| Bernal et al. (2013) | Observational study | - | - | - | X | X | - | Impact on wages; Education/ Cognitive development assessed with Early Development Instrument (EDI) | - | X | - | - |
| Bhutta et al. (2013) | Previously published studies | X | X | - | X | - | - | - | X | X | DT | Lives Saved Tool (LiST) |
| Chola et al. (2015) | RCT | X | - | DALY | X | - | - | - | X | - | DT+MM | Original |
| Chow et al. (2010) | Previously published studies and assumptions | X | - | DALY | X | X | - | - | X | X | DT | Zimmerman and Qaim (2004) |
| Danielsson et al. (2004) | Observational study | X | X | DALY | X | - | - | - | X | X | DT | Original |
| Desmond et al. (2008) | Observational study and assumptions | - | - | - | X | - | - | - | X | - | MM | Original |
| Edejer et al. (2005) | Meta-analysis | X | - | DALY | X | - | Measles, pneumonia | - | X | X | DT | Original |
| Feldhaus et al. (2016) | Observational study | X | - | DALY | - | - | - | Lost wages | X | X | DT | Original |
| Fink et al. (2014) | Previously published studies | X | - | DALY | X | - | - | - | N/S | X | DT | Stein (2006) |
| Goudet et al. (2018a) | RCT | - | - | - | - | X | - | School performance; Cognitive development; Labour participation and impact on income due to job loss | X | X | - | - |
| Lopez Boo et al. (2014) | Quasi-experiment | - | - | - | X | X | - | Years of education; Impact on wages/earnings; Education/cognitive outcomes measured by the McCarthy Scales of Children’s Ability (MSCA) | X | - | - | - |
| Ma et al. (2008) | N/S | - | - | DALY | - | - | - | - | - | X | N/S | N/S |
| Qureshy et al. (2013) | Previously published studies and assumptions | X | - | - | X | X | - | Productivity enhancement  and productive earnings | X | X | - | - |
| Schreinemachers et al. (2016) | Quasi-experiment and previously published studies | - | - | DALY | - | - | Vegetables consumed | Vegetable production | - | X | - | - |
| Shaheen et al. (2015) | RCT | X | X | - | - | - | - | Lost productivity | X | X | - | - |
| Sharieff et al. (2006) | Previously published studies and assumptions | X | - | DALY | X | - | IQ | Cognitive achievement;  Gain in earnings | - | X | DT | Original |
| Shekar et al. (2016) | Literature review | X | - | X | X | - | - | - | X | X | DT | Lives Saved Tool (LiST) |
| Svefors et al. (2018) | RCT | X | X | DALY | X | - | - | - | X | X | DT | Original |
| Walters et al. (2016) | Quasi-experiment and assumptions | X | - | - | X | - | - | Earnings; cognitive development | X | X | - | - |
| Waters et al. (2006) | RCT and previously published studies | X | - | - | X | - | - | - | X | X | - | - |

YLL: Years of Life Lost; DALY: Disability-Adjusted Life Year; HS: Health System; N/S: Not specified or not clear; DT: Decision Tree; MM: Markov Model

| **2. Therapeutic nutrition interventions** | | | | | | | | | | | | |
| --- | --- | --- | --- | --- | --- | --- | --- | --- | --- | --- | --- | --- |
| **Authors** | **Method for the estimation of the treatment effect** | **Health Outcomes** | | | | | | **Non-health outcomes** | **Costs** | | **Model** | |
|  |  | **Mortality** | **YLL** | **Generic** | **Disease specific** | **Monetary values** | **Other** |  | **HS** | **Non-HS** | **Type** | **Reference** |
| Akram et al. (2016) | Observational study | - | - | - | X | - | - | - | X | X | - | - |
| Bachmann (2009) | Previously published studies and observational study | X | - | X | - | - | - | - | X | - | DT | Original |
| Bhutta et al. (2013) | Previously published studies | X | DALY | - | X | - | - | - | X | X | DT | Lives Saved Tool (LiST) |
| Goudet et al. (2018b) | Quasi-experiment | X | DALY | X | - | - | - | - | X | X | DT | Original |
| Mejı´a et al. (2015) | Previously published studies | X | - | - | X | - | - | - | X | - | DT | Original |
| Puett et al. (2013) | Observational study | X | DALY | X | X | - | - | - | X | X | DT | Original |
| Robberstad et al. (2004) | Previously published studies | X | - | X | X | - | - | - | X | X | DT | Original |
| Rogers et al. (2018) | RCT | X | - | - | X | - | - | Forgone income | X | X | - | - |
| Rogers et al. (2019) | RCT | X | - | - | X | - | - | Lost income | X | X | - | - |
| Shekar et al. (2016) | Literature review | X | - | X | X | - | - | - | X | X | DT | Lives Saved Tool (LiST) |
| Shillcutt et al. (2017) | Observational study | - | - | - | X | X | - | Wages lost | X | X | - | - |
| Wilford et al. (2012) | Observational study and assumptions | X | DALY | X | - | - | - | - | X | X | DT | Bachmann (2009) |

YLL: Years of Life Lost; DALY: Disability-Adjusted Life Year; HS: Health System; N/S: Not specified or not clear; DT: Decision Tree; MM: Markov Model

| **3. Fortification strategies** | | | | | | | | | | | | |
| --- | --- | --- | --- | --- | --- | --- | --- | --- | --- | --- | --- | --- |
| **Authors** | **Method for the estimation of the treatment effect** | **Health Outcomes** | | | | | | **Non-health outcomes** | **Costs** | | **Model** | |
|  |  | **Mortality** | **YLL** | **Generic** | **Disease specific** | **Monetary values** | **Other** |  | **HS** | **Non-HS** | **Type** | **Reference** |
| Baltussen et al. (2004) | Previously published studies and assumptions | X | - | DALY | - | - | - | - | X | X | MM | Original |
| Bhutta et al. (2013) | Previously published studies | X | X | - | X | - | - | - | X | X | DT | Lives Saved Tool (LiST) |
| Chow et al. (2010) | Literature review, assumptions and expert opinion | X | - | DALY | X | X | - | - | - | X | DT | Zimmerman and Qaim (2004) |
| Dainelli et al. (2017) | RCT | X | - | QALY | X | - | - | Days of work lost | X | X | MM | Original |
| De Steur et al. (2012a) | Previously published studies and assumptions | X | X | DALY | X | - | - | - | - | X | DT | HarvestPlus (2005) |
| De Steur et al. (2012b) | Previously published studies and assumptions | X | X | DALY | X | - | - | - | - | X | DT | HarvestPlus (2005) |
| Edejer et al. (2005) | Meta-analysis | X | - | DALY | X | - | Measles, pneumonia | - | X | X | DT | Original |
| Fiedler et al. (2009) | Assumptions | - | - | DALY | - | - | - | - | - | X | DT | HarvestPlus (2005) |
| Fiedler et al. (2010) | Assumptions | X | X | DALY | X | - | - | - | - | X | DT | HarvestPlus (2005) |
| Fiedler et al. (2013) | Assumptions | - | - | DALY | - | - | - | - | - | X | DT | (Modified) HarvestPlus (2005) |
| Fiedler et al. (2015) | Assumptions | X | X | DALY | - | - | - | - | - | X | DT | (Modified) HarvestPlus (2005) |
| Fiedler et al. (2016) | Assumptions | X | - | DALY | - | X | - | - | - | X | DT | HarvestPlus (2005) |
| Hoddinott (2018) | Previously published studies | X | - | DALY | X | - | - | - | - | X | DT | Original |
| Lividini et al. (2015) | Assumptions | - | - | DALY | - | X | - | - | - | X | DT | HarvestPlus (2005) |
| Ma et al. (2008) | N/S | - | - | DALY | - | - | - | - | - | X | N/S | N/S |
| Meenakshi et al. (2010) | Previously published studies and assumptions | - | - | DALY | - | - | - | - | - | X | DT | HarvestPlus (2005) |
| Shekar et al. (2016) | Literature review | X | - | X | X | - | - | - | X | X | DT | Lives Saved Tool (LiST) |
| Stein et al. (2006) | Assumptions | X | - | DALY | - | - |  | - | - | X | DT | HarvestPlus (2005) |
| Stein et al. (2007) | Assumptions | X | - | DALY | X | X | - | - | - | X | DT | HarvestPlus (2005) |
| Stein et al. (2008) | Previously published studies and assumptions | X | - | DALY | - | - | - | - | - | X | DT | HarvestPlus (2005) |
| Wang et al. (2016) | Previously published studies and assumptions | - | - | DALY | - | - | - | - | - | X | DT | HarvestPlus (2005) |
| Zhang et al. (2018) | Previously published studies | - | - | DALY | - | - | - | - | - | X | DT | De Steur et al. (2012a); Zimmerman and Qaim (2004) |

YLL: Years of Life Lost; DALY: Disability-Adjusted Life Year; HS: Health System; N/S: Not specified or not clear; DT: Decision Tree; MM: Markov Model

| **4. Delivery platforms** | | | | | | | | | | | | |
| --- | --- | --- | --- | --- | --- | --- | --- | --- | --- | --- | --- | --- |
| **Authors** | **Method for the estimation of the treatment effect** | **Health Outcomes** | | | | | | **Non-health outcomes** | **Costs** | | **Model** | |
|  |  | **Mortality** | **YLL** | **Generic** | **Disease specific** | **Monetary values** | **Other** |  | **HS** | **Non-HS** | **Type** | **Reference** |
| Bergmann et al. (2017) | Quasi-experiment | X | - | DALY | X | - | HIV infections | - | X | X | - | - |
| Bishai et al. (2015) | Quasi-experiment | X | - | DALY | X | - | - | Loss of wages | X | X | DT | Original |
| Plessow et al. (2016) | Hypothetical buying experiment | X | X | DALY | - | - | - | Production losses and lower future income | - | X | DT | Original |
| Trenouth et al. (2018) | RCT | X | X | DALY | X | - | - | - | N/S | X | DT | Original |
| Wieser et al. (2018) | Market survey | X | X | DALY | - | - | - | Production losses | X | X | DT | Plessow et al. (2016) |

YLL: Years of Life Lost; DALY: Disability-Adjusted Life Year; HS: Health System; N/S: Not specified or not clear; DT: Decision Tree; MM: Markov Model

## Table S3: Quality assessment – CHEERS Checklist

| **Section/item** | **Satisfied** | **Partially Satisfied** | **Not Satisfied** | **Not Applicable** |
| --- | --- | --- | --- | --- |
| Title and abstract |  |  |  |  |
| Title | 37 (69%) | 6 (11%) | 11 (20%) | 0 |
| Abstract | 48 (89%) | 5 (9%) | 1 (2%) | 0 |
| Introduction |  |  |  |  |
| Background and objectives | 51 (94%) | 2 (4%) | 1 (2%) | 0 |
| Methods |  |  |  |  |
| Target population and subgroups | 47 (87%) | 6 (11%) | 1 (2%) | 0 |
| Setting and location | 50 (93%) | 2 (4%) | 2 (4%) | 0 |
| Study perspective | 19 (35%) | 4 (7%) | 31 (57%) | 0 |
| Comparators | 45 (83%) | 8 (15%) | 1 (2%) | 0 |
| Time horizon | 37 (69%) | 2 (4%) | 15 (28%) | 0 |
| Discount rate | 31 (57%) | 2 (4%) | 21 (39%) | 0 |
| Choice of health outcomes | 44 (81%) | 6 (11%) | 4 (7%) | 0 |
| Measurement of effectiveness | 46 (85%) | 5 (9%) | 3 (6%) | 0 |
| Measurement and valuation of preference based outcomes | 22 (55%) | 14 (35%) | 4 (10%) | 14 |
| Estimating resources and costs | 41 (76%) | 8 (15%) | 5 (9%) | 0 |
| Currency, price date, and conversion | 34 (63%) | 6 (11%) | 14 (26%) | 0 |
| Choice of model | 17 (44%) | 10 (26%) | 12 (31%) | 15 |
| Assumptions | 43 (83%) | 5 (10%) | 4 (8%) | 2 |
| Analytical methods | 35 (65%) | 13 (24%) | 6 (11%) | 0 |
| Results |  |  |  |  |
| Study parameters | 40 (74%) | 7 (13%) | 7 (13%) | 0 |
| Incremental costs and outcomes | 49 (91%) | 3 (6%) | 2 (4%) | 0 |
| Characterising uncertainty | 38 (70%) | 3 (6%) | 13 (24%) | 0 |
| Characterising heterogeneity | 19 (79%) | 4 (17%) | 1 (4%) | 30 |
| Discussion |  |  |  |  |
| Study findings, limitations, generalisability, and current knowledge | 41 (76%) | 13 (24%) | 0 (0%) | 0 |
| Other |  |  |  |  |
| Source of funding | 46 (85%) | 2 (4%) | 6 (11%) | 0 |
| Conflicts of interest | 37 (69%) | 0 (0%) | 17 (31%) | 0 |
| **Items satisfied or partially satisfied overall** | **85%** | | | |
| * Percentages refer only to studies where the item is applicable |  |  |  |  |

| **Section/item** | **Preventive** | **Therapeutic** | **Fortification** | **Delivery Platform** |
| --- | --- | --- | --- | --- |
| Title and abstract |  |  |  |  |
| Title | 70% | 75% | 77% | 100% |
| Abstract | 100% | 100% | 95% | 100% |
| Introduction |  |  |  |  |
| Background and objectives | 96% | 100% | 100% | 100% |
| Methods |  |  |  |  |
| Target population and subgroups | 96% | 92% | 95% | 100% |
| Setting and location | 91% | 92% | 91% | 100% |
| Study perspective | 22% | 75% | 23% | 80% |
| Comparators | 100% | 100% | 95% | 100% |
| Time horizon | 70% | 75% | 73% | 40% |
| Discount rate | 57% | 75% | 59% | 40% |
| Choice of health outcomes | 96% | 83% | 95% | 100% |
| Measurement of effectiveness | 91% | 92% | 86% | 100% |
| Measurement and valuation of preference based outcomes | 86% | 86% | 91% | 60% |
| Estimating resources and costs | 91% | 92% | 91% | 100% |
| Currency, price date, and conversion | 78% | 92% | 64% | 60% |
| Choice of model | 58% | 78% | 68% | 100% |
| Assumptions | 86% | 92% | 95% | 100% |
| Analytical methods | 87% | 83% | 86% | 80% |
| Results |  |  |  |  |
| Study parameters | 87% | 92% | 86% | 80% |
| Incremental costs and outcomes | 96% | 92% | 100% | 100% |
| Characterising uncertainty | 65% | 83% | 77% | 80% |
| Characterising heterogeneity | 91% | 75% | 93% | 100% |
| Discussion |  |  |  |  |
| Study findings, limitations, generalisability, and current knowledge | 100% | 100% | 100% | 100% |
| Other |  |  |  |  |
| Source of funding | 100% | 92% | 77% | 100% |
| Conflicts of interest | 74% | 83% | 55% | 100% |
| **Items satisfied or partially satisfied overall** | **83%** | **88%** | **82%** | **88%** |
| * Percentages refer only to studies where the item is applicable | | | | |

## Table S4: Non-Health outcomes

| **Impacts on:** | **Number of studies** | | | | |
| --- | --- | --- | --- | --- | --- |
|  | **Total** | **Preventive** | **Therapeutic** | **Fortification** | **Delivery** |
| Productivity | 11 | 4 | 3 | 1 | 3 |
| Productivity and education | 5 | 5 | 0 | 0 | 0 |

## Table S5: Studies addressing specific micronutrient deficiencies

The table below reports studies that directly compare strategies in different topic areas, together with additional studies that investigate single strategies for the four most common micro-nutrient deficiencies (i.e. vitamin A, zinc, iron and folic acid).

|  | **Vitamin A** | **Iron** | **Zinc** | **Folic acid** |
| --- | --- | --- | --- | --- |
| *Studies that compare alternative strategies* | | | | |
| **Preventive & Fortification** | Chow et al. (2010)  Edejer et al. (2005) | Baltussen et al. (2004)  Ma et al. (2008) | Edejer et al. (2005)  Ma et al. (2008) | Bhutta et al. (2013) |
| *Studies that investigate a single strategy* | | | | |
| **Preventive** | Bhutta et al. (2013) | Alonzo Gonzalez et al. (2000)  Shekar et al. (2016) | Bhutta et al. (2013)  Fink et al. (2014) | Shekar et al. (2016) |
| **Fortification** | Fiedler et al. (2010)  Fiedler et al. (2016)  Lividini et al. (2015)  Stein et al. (2006) | Stein et al. (2008)  Zhang et al. (2018)  Shekar et al. (2016) | Stein et al. (2007)  Wang et al. (2016)  Zhang et al. (2018) | De Steur et al. (2012b)  Hoddinott (2018) |
| **Therapeutic** | - | - | Mejı´a et al. (2015)  Robberstad et al. (2004)  Shillcutt et al. (2017)  Shekar et al. (2016) | - |
| **Delivery** | - | Plessow et al. (2016) | Bishai et al. (2015) | - |

## Table S6: Cost-effectiveness estimates of strategies to address zinc deficiency

| **Costs (currency year)** | **Outcomes** | **Comparator** | **Country/Region** | **Authors (year)** |
| --- | --- | --- | --- | --- |
| **Preventive interventions** | | | | |
| I$399 (not stated) | Not clear | Not clear | China | (Ma et al., 2008) |
| I$48 (2000) | DALY averted | Doing nothing | South East Asia | (Edejer et al., 2005) |
| I$122 (2000) | DALY averted | Doing nothing | Sub-Saharan Africa | (Edejer et al., 2005) |
| US$ 606-1211 (not stated) | DALY averted | Doing nothing | Representative low-income country | (Fink and Heitner, 2014) |
| **Fortification strategies** | | | | |
| I$153 (not stated) | Not clear | Not clear | China | (Ma et al., 2008) |
| US$226-594 (not stated) | DALY averted | Doing nothing | China | (Wang et al., 2016) |
| US$311-4989 (2015) | DALY averted | Doing nothing | China | (Zhang et al., 2018) |
| I$14 (2000) | DALY averted | Doing nothing | South East Asia | (Edejer et al., 2005) |
| I$55 (2000) | DALY averted | Doing nothing | Sub-Saharan Africa | (Edejer et al., 2005) |
| US$0.73-7.31 (2004) | DALY averted | Doing nothing | India | (Stein et al., 2007) |
| **Therapeutic interventions** | | | | |
| $5.50-$7.50 (2014) | Diarrhoea episode | Doing nothing | India | (Shillcutt et al., 2017) |
| Dominant (2010) | Diarrhoea episode; death | Standard treatment | Colombia | (Mejia et al., 2015) |
| US$ 40 (2001) | DALY averted | Standard treatment | United Republic of Tanzania | (Robberstad et al., 2004) |
| $71 (2015), $41 (2015), $84 (2014), $59 (2015) | DALY averted | Current coverage | DRC, Mali, Nigeria, Togo | (Shekar et al., 2016) |
| **Delivery platforms** | | | | |
| Societal perspective: $214 (2010)  Medical perspective: $339 (2010) | DALY averted | Standard practices | Myanmar | (Bishai et al., 2015) |

## Search strategies

### MEDLINE

Database: Ovid MEDLINE(R) ALL <1946 to March 27, 2019>

Search Strategy:

--------------------------------------------------------------------------------

1 exp Malnutrition/ (116782)

2 Protein-energy Malnutrition/ (7192)

3 exp Severe Acute Malnutrition/ (2732)

4 (malnutrition or malnourish$).ti,ab,kw. (41114)

5 (undernutrition or under-nutrition).ti,ab,kw. (7409)

6 ((child$ or infancy or infant$ or maternal or mother$) adj2 nutrition).ti,ab,kw. (9529)

7 Food Assistance/ (766)

8 ((feeding or food or hunger or malnutrition or nutrition) adj program$).ti,ab,kw. (3999)

9 ((feeding or food or hunger or malnutrition or nutrition) adj (assist$ or help)).ti,ab,kw. (940)

10 food aid.ti,ab,kw. (280)

11 exp Nutrition Disorders/ (331321)

12 Infant Nutrition Disorders/ (4515)

13 exp Deficiency Diseases/ (94319)

14 Anemia, Iron Deficiency/ (9208)

15 Kwashiorkor/ (2599)

16 Fortified Foods/ (8949)

17 (food adj2 (fortif$ or biofortif$ or supplementary)).ti,ab,kw. (1898)

18 Fast Foods/ (1786)

19 Dietary Supplements/ (51142)

20 (supplement$ adj2 (calcium or folate or folic or iodine or iron or micronutrient$ or vitamin$ or zinc)).ti,ab,kw. (37348)

21 Calcium, Dietary/ (13755)

22 Folic Acid/ (25985)

23 Iodine/ (24591)

24 Vitamin A/ (23501)

25 Vitamin K/ (11300)

26 Micronutrients/ (5240)

27 Iron, Dietary/ (2814)

28 Zinc/ (57346)

29 Maternal Nutritional Physiological Phenomenon/ (3798)

30 Infant Food/ (9746)

31 Breast Feeding/ (35258)

32 (breastfeed$ or breastfed$).ti,ab,kw. (25987)

33 Rehydration Solutions/ (1423)

34 Oral rehydrat$ salt$.ti,ab,kw. (377)

35 Anthelmintics/ (14228)

36 deworm$.ti,ab,kw. (1242)

37 ("ready to use food" or "ready to use therapeutic food" or "ready to use supplementary food" or RUT or RUTF or RUSF).ti,ab,kw. (1162)

38 (plumpynut or plumpy'nut or plumpysoy or nutributter).ti,ab,kw. (25)

39 or/1-38 (632483)

40 Global Health/ (42457)

41 Developing Countries.sh,kf. (82965)

42 (Africa or Asia or Caribbean or West Indies or South America or Latin America or Central America).hw,kf,ti,ab,cp. (254946)

43 (Afghanistan or Albania or Algeria or Angola or Antigua or Barbuda or Argentina or Armenia or Armenian or Aruba or Azerbaijan or Bahrain or Bangladesh or Barbados or Benin or Byelarus or Byelorussian or Belarus or Belorussian or Belorussia or Belize or Bhutan or Bolivia or Bosnia or Herzegovina or Hercegovina or Botswana or Brasil or Brazil or Bulgaria or Burkina Faso or Burkina Fasso or Upper Volta or Burundi or Urundi or Cambodia or Khmer Republic or Kampuchea or Cameroon or Cameroons or Cameron or Camerons or Cape Verde or Central African Republic or Chad or Chile or China or Colombia or Comoros or Comoro Islands or Comores or Mayotte or Congo or Zaire or Costa Rica or Cote d'Ivoire or Ivory Coast or Croatia or Cuba or Cyprus or Czechoslovakia or Czech Republic or Slovakia or Slovak Republic or Djibouti or French Somaliland or Dominica or Dominican Republic or East Timor or East Timur or Timor Leste or Ecuador or Egypt or United Arab Republic or El Salvador or Eritrea or Estonia or Ethiopia or Fiji or Gabon or Gabonese Republic or Gambia or Gaza or Georgia Republic or Georgian Republic or Ghana or Gold Coast or Greece or Grenada or Guatemala or Guinea or Guam or Guiana or Guyana or Haiti or Honduras or Hungary or India or Maldives or Indonesia or Iran or Iraq or Isle of Man or Jamaica or Jordan or Kazakhstan or Kazakh or Kenya or Kiribati or Korea or Kosovo or Kyrgyzstan or Kirghizia or Kyrgyz Republic or Kirghiz or Kirgizstan or Lao PDR or Laos or Latvia or Lebanon or Lesotho or Basutoland or Liberia or Libya or Lithuania or Macedonia or Madagascar or Malagasy Republic or Malaysia or Malaya or Malay or Sabah or Sarawak or Malawi or Nyasaland or Mali or Malta or Marshall Islands or Mauritania or Mauritius or Agalega Islands or Mexico or Micronesia or Middle East or Moldova or Moldovia or Moldovian or Mongolia or Montenegro or Morocco or Ifni or Mozambique or Myanmar or Myanma or Burma or Namibia or Nepal or Netherlands Antilles or New Caledonia or Nicaragua or Niger or Nigeria or Northern Mariana Islands or Oman or Muscat or Pakistan or Palau or Palestine or Panama or Paraguay or Peru or Philippines or Philipines or Phillipines or Phillippines or Poland or Portugal or Puerto Rico or Romania or Rumania or Roumania or Russia or Russian or Rwanda or Ruanda or Saint Kitts or St Kitts or Nevis or Saint Lucia or St Lucia or Saint Vincent or St Vincent or Grenadines or Samoa or Samoan Islands or Navigator Island or Navigator Islands or Sao Tome or Saudi Arabia or Senegal or Serbia or Montenegro or Seychelles or Sierra Leone or Slovenia or Sri Lanka or Ceylon or Solomon Islands or Somalia or South Africa or Sudan or Suriname or Surinam or Swaziland or Syria or Tajikistan or Tadzhikistan or Tadjikistan or Tadzhik or Tanzania or Thailand or Togo or Togolese Republic or Tonga or Trinidad or Tobago or Tunisia or Turkey or Turkmenistan or Turkmen or Uganda or Ukraine or Uruguay or USSR or Soviet Union or Union of Soviet Socialist Republics or Uzbekistan or Uzbek or Vanuatu or New Hebrides or Venezuela or Vietnam or Viet Nam or West Bank or Yemen or Yugoslavia or Zambia or Zimbabwe or Rhodesia).hw,kf,ti,ab,cp. (3460354)

44 ((developing or less* developed or under developed or underdeveloped or middle income or low* income or underserved or under served or deprived or poor*) adj (countr* or nation? or population? or world)).ti,ab. (89128)

45 ((developing or less* developed or under developed or underdeveloped or middle income or low* income) adj (economy or economies)).ti,ab. (472)

46 (low* adj (gdp or gnp or gross domestic or gross national)).ti,ab. (226)

47 (low adj3 middle adj3 countr*).ti,ab. (12481)

48 (lmic or lmics or third world or lami countr*).ti,ab. (6227)

49 transitional countr*.ti,ab. (153)

50 or/40-49 (3631873)

51 Economics/ (27016)

52 exp "Costs and Cost Analysis"/ (223171)

53 Economics, Nursing/ (3986)

54 Economics, Medical/ (9007)

55 Economics, Pharmaceutical/ (2852)

56 exp Economics, Hospital/ (23439)

57 Economics, Dental/ (1902)

58 exp "Fees and Charges"/ (29658)

59 exp Budgets/ (13477)

60 budget*.ti,ab,kf. (27224)

61 (economic* or cost or costs or costly or costing or price or prices or pricing or pharmacoeconomic* or pharmaco-economic* or expenditure or expenditures or expense or expenses or financial or finance or finances or financed).ti,kf. (210457)

62 (economic* or cost or costs or costly or costing or price or prices or pricing or pharmacoeconomic* or pharmaco-economic* or expenditure or expenditures or expense or expenses or financial or finance or finances or financed).ab. /freq=2 (259292)

63 (cost* adj2 (effective* or utilit* or benefit* or minimi* or analy* or outcome or outcomes)).ab,kf. (145228)

64 (value adj2 (money or monetary)).ti,ab,kf. (2147)

65 exp models, economic/ (13961)

66 economic model*.ab,kf. (2979)

67 markov chains/ (13298)

68 markov.ti,ab,kf. (20162)

69 monte carlo method/ (26531)

70 monte carlo.ti,ab,kf. (45160)

71 exp Decision Theory/ (11388)

72 (decision* adj2 (tree* or analy* or model*)).ti,ab,kf. (20817)

73 (ROI or "return on investment").ti,ab,kw. (10560)

74 or/51-73 (681128)

75 39 and 50 and 74 (5656)

***************************

### Embase

Database: Embase <1974 to 2019 March 29>

Search Strategy:

--------------------------------------------------------------------------------

1 exp Malnutrition/ (144365)

2 Protein Deficiency/ (14754)

3 (malnutrition or malnourish$).ti,ab,kw. (55655)

4 (undernutrition or under-nutrition).ti,ab,kw. (9482)

5 ((child$ or infancy or infant$ or maternal or mother$) adj2 nutrition).ti,ab,kw. (9251)

6 Food Assistance/ (1156)

7 ((feeding or food or hunger or malnutrition or nutrition) adj program$).ti,ab,kw. (4311)

8 ((feeding or food or hunger or malnutrition or nutrition) adj (assist$ or help)).ti,ab,kw. (1165)

9 food aid.ti,ab,kw. (315)

10 exp Nutritional Disorder/ (804482)

11 exp Nutritional Deficiency/ (132223)

12 Iron Deficiency/ (14837)

13 Kwashiorkor/ (2584)

14 Fortified Food/ (804)

15 (food adj2 (fortif$ or biofortif$ or supplementary)).ti,ab,kw. (2525)

16 Dietary Supplementation/ (81443)

17 (supplement$ adj2 (calcium or folate or folic or iodine or iron or micronutrient$ or vitamin$ or zinc)).ti,ab,kw. (52476)

18 Calcium Deficiency/ (1571)

19 Folic Acid Deficiency/ (6418)

20 Iodine Deficiency/ (4414)

21 Vitamin Deficiency/ (8811)

22 Retinol Deficiency/ (5926)

23 Vitamin K Deficiency/ (2140)

24 Mineral Deficiency/ (776)

25 Iron Deficiency/ (14837)

26 Zinc Deficiency/ (6034)

27 Maternal Nutrition/ (10902)

28 exp Infant Nutrition/ (82472)

29 Breast Feeding/ (47640)

30 (breastfeed$ or breastfed$).ti,ab,kw. (31882)

31 Oral Rehydration Solution/ (2825)

32 Oral rehydrat$ salt$.ti,ab,kw. (393)

33 Anthelmintic Agent/ (8984)

34 deworm$.ti,ab,kw. (1530)

35 ("ready to use food" or "ready to use therapeutic food" or "ready to use supplementary food" or RUT or RUTF or RUSF).ti,ab,kw. (1567)

36 (plumpynut or plumpy'nut or plumpysoy or nutributter).ti,ab,kw. (33)

37 1 or 2 or 3 or 4 or 5 or 6 or 7 or 8 or 9 or 10 or 11 or 12 or 13 or 14 or 15 or 16 or 17 or 18 or 19 or 20 or 21 or 22 or 23 or 24 or 25 or 26 or 27 or 28 or 29 or 30 or 31 or 32 or 33 or 34 or 35 or 36 (1011573)

38 Developing Country.sh. (90200)

39 (Africa or Asia or Caribbean or West Indies or South America or Latin America or Central America).hw,ti,ab,cp. (314309)

40 (Afghanistan or Albania or Algeria or Angola or Antigua or Barbuda or Argentina or Armenia or Armenian or Aruba or Azerbaijan or Bahrain or Bangladesh or Barbados or Benin or Byelarus or Byelorussian or Belarus or Belorussian or Belorussia or Belize or Bhutan or Bolivia or Bosnia or Herzegovina or Hercegovina or Botswana or Brasil or Brazil or Bulgaria or Burkina Faso or Burkina Fasso or Upper Volta or Burundi or Urundi or Cambodia or Khmer Republic or Kampuchea or Cameroon or Cameroons or Cameron or Camerons or Cape Verde or Central African Republic or Chad or Chile or China or Colombia or Comoros or Comoro Islands or Comores or Mayotte or Congo or Zaire or Costa Rica or Cote d'Ivoire or Ivory Coast or Croatia or Cuba or Cyprus or Czechoslovakia or Czech Republic or Slovakia or Slovak Republic or Djibouti or French Somaliland or Dominica or Dominican Republic or East Timor or East Timur or Timor Leste or Ecuador or Egypt or United Arab Republic or El Salvador or Eritrea or Estonia or Ethiopia or Fiji or Gabon or Gabonese Republic or Gambia or Gaza or Georgia Republic or Georgian Republic or Ghana or Gold Coast or Greece or Grenada or Guatemala or Guinea or Guam or Guiana or Guyana or Haiti or Honduras or Hungary or India or Maldives or Indonesia or Iran or Iraq or Isle of Man or Jamaica or Jordan or Kazakhstan or Kazakh or Kenya or Kiribati or Korea or Kosovo or Kyrgyzstan or Kirghizia or Kyrgyz Republic or Kirghiz or Kirgizstan or Lao PDR or Laos or Latvia or Lebanon or Lesotho or Basutoland or Liberia or Libya or Lithuania or Macedonia or Madagascar or Malagasy Republic or Malaysia or Malaya or Malay or Sabah or Sarawak or Malawi or Nyasaland or Mali or Malta or Marshall Islands or Mauritania or Mauritius or Agalega Islands or Mexico or Micronesia or Middle East or Moldova or Moldovia or Moldovian or Mongolia or Montenegro or Morocco or Ifni or Mozambique or Myanmar or Myanma or Burma or Namibia or Nepal or Netherlands Antilles or New Caledonia or Nicaragua or Niger or Nigeria or Northern Mariana Islands or Oman or Muscat or Pakistan or Palau or Palestine or Panama or Paraguay or Peru or Philippines or Philipines or Phillipines or Phillippines or Poland or Portugal or Puerto Rico or Romania or Rumania or Roumania or Russia or Russian or Rwanda or Ruanda or Saint Kitts or St Kitts or Nevis or Saint Lucia or St Lucia or Saint Vincent or St Vincent or Grenadines or Samoa or Samoan Islands or Navigator Island or Navigator Islands or Sao Tome or Saudi Arabia or Senegal or Serbia or Montenegro or Seychelles or Sierra Leone or Slovenia or Sri Lanka or Ceylon or Solomon Islands or Somalia or South Africa or Sudan or Suriname or Surinam or Swaziland or Syria or Tajikistan or Tadzhikistan or Tadjikistan or Tadzhik or Tanzania or Thailand or Togo or Togolese Republic or Tonga or Trinidad or Tobago or Tunisia or Turkey or Turkmenistan or Turkmen or Uganda or Ukraine or Uruguay or USSR or Soviet Union or Union of Soviet Socialist Republics or Uzbekistan or Uzbek or Vanuatu or New Hebrides or Venezuela or Vietnam or Viet Nam or West Bank or Yemen or Yugoslavia or Zambia or Zimbabwe or Rhodesia).hw,ti,ab,cp. (3753234)

41 ((developing or less* developed or under developed or underdeveloped or middle income or low* income or underserved or under served or deprived or poor*) adj (countr* or nation? or population? or world)).ti,ab. (111804)

42 ((developing or less* developed or under developed or underdeveloped or middle income or low* income) adj (economy or economies)).ti,ab. (612)

43 (low* adj (gdp or gnp or gross domestic or gross national)).ti,ab. (331)

44 (low adj3 middle adj3 countr*).ti,ab. (14365)

45 (lmic or lmics or third world or lami countr*).ti,ab. (7454)

46 transitional countr*.ti,ab. (219)

47 38 or 39 or 40 or 41 or 42 or 43 or 44 or 45 or 46 (3954844)

48 37 and 47 (166654)

49 Economics/ (231841)

50 exp "Costs and Cost Analysis"/ (327863)

51 Economics, Nursing/ (29776)

52 Economics, Medical/ (30683)

53 Economics, Pharmaceutical/ (6982)

54 exp Economics, Hospital/ (787380)

55 Economics, Dental/ (31674)

56 exp "Fees and Charges"/ (38397)

57 exp Budgets/ (26938)

58 budget*.ti,ab,kf. (35017)

59 (economic* or cost or costs or costly or costing or price or prices or pricing or pharmacoeconomic* or pharmaco-economic* or expenditure or expenditures or expense or expenses or financial or finance or finances or financed).ti,kf. (223625)

60 (economic* or cost or costs or costly or costing or price or prices or pricing or pharmacoeconomic* or pharmaco-economic* or expenditure or expenditures or expense or expenses or financial or finance or finances or financed).ab. /freq=2 (360383)

61 (cost* adj2 (effective* or utilit* or benefit* or minimi* or analy* or outcome or outcomes)).ab,kf. (198246)

62 (value adj2 (money or monetary)).ti,ab,kf. (2950)

63 exp models, economic/ (1584)

64 economic model*.ab,kf. (4060)

65 markov chains/ (3641)

66 markov.ti,ab,kf. (24797)

67 monte carlo method/ (35402)

68 monte carlo.ti,ab,kf. (42253)

69 exp Decision Theory/ (1689)

70 (decision* adj2 (tree* or analy* or model*)).ti,ab,kf. (28823)

71 (ROI or "return on investment").ti,ab,kw. (19014)

72 or/49-71 (1301051)

73 48 and 72 (9516)

74 limit 73 to embase (5684)

***************************

<1>

AU - Efunshile A.M.

AU - Ezeanosike O.

AU - Nwangwu C.C.

AU - Konig B.

AU - Jokelainen P.

AU - Robertson L.J.

TI - Apparent overuse of antibiotics in the management of watery diarrhoea in children in Abakaliki, Nigeria.

SO - BMC Infectious Diseases. 19 (1) (no pagination), 2019. Article Number: 275. Date of Publication: 21 Mar 2019.

PB - BioMed Central Ltd. (E-mail: info@biomedcentral.com)

PT - Article

## Extraction table

| **First author** |  | | | | | | | | | | | | | | | | | | | |
| --- | --- | --- | --- | --- | --- | --- | --- | --- | --- | --- | --- | --- | --- | --- | --- | --- | --- | --- | --- | --- |
| **Year** |  | | | | | | | | | | | | | | | | | | | |
| **Country** |  | | | | | | | | | | | | | | | | | | | |
| **Target population** |  | | | | | | | | | | | | | | | | | | | |
| **Intervention** |  | | | | | | | | | | | | | | | | | | | |
| **Comparator** |  | | | | | | | | | | | | | | | | | | | |
| **Type of study** | CEA |  |  | | | | | | | | | | | | | | | | | |
|  | CBA |  |  | | | | | | | | | | | | | | | | | |
|  | Other |  | *Specify^[[1]](#footnote-1)^* | | | | | | | | | | | | | | | | | |
| **Nutritional deficiencies treated** | Iron |  | | Iodine | | | | | | | | | | |  | |  | | | |
|  | Vitamin A |  | | Vitamin K | | | | | | | | | | |  | |  | | | |
|  | Zinc |  | | Protein – energy | | | | | | | | | | |  | |  | | | |
|  | Folate |  | | Other | | | | | | | | | | |  | | *Specify* | | | |
| **Method for the estimation of the treatment effect^[[2]](#footnote-2)^** | RCT | | | |  | |  | | | | | | | | | | | | | |
|  | Quasi-experiment^[[3]](#footnote-3)^ | | | |  | |  | | | | | | | | | | | | | |
|  | Literature review | | | |  | |  | | | | | | | | | | | | | |
|  | Other | | | |  | | *Specify^[[4]](#footnote-4)^* | | | | | | | | | | | | | |
| **Health outcomes** | Mortality | | | | | | |  | | | | *Details^[[5]](#footnote-5)^* | | | | | | | | |
|  | Life years lost | | | | | | |  | | | |  | | | | | | | | |
|  | Generic (QALYs, DALYs) | | | | | | |  | | | | *Details^[[6]](#footnote-6)^* | | | | | | | | |
|  | Disease specific | | | | | | |  | | | | *Details^[[7]](#footnote-7)^* | | | | | | | | |
|  | Monetary values | | | | | | |  | | | |  | | | | | | | | |
|  | Other | | | | | | |  | | | | *Specify* | | | | | | | | |
| **Modelling^[[8]](#footnote-8)^** | Study does not include model | | | | | | | | |  | | |  | | | | | | | |
|  | Study includes a model | | | | | | | | |  | | | *If yes, specify:* | | | | | | | |
|  | Type: | | Markov model | | | | | | | |  | | | | | Decision tree | |  | Other |  |
|  | Population: | | Cohort | | | | | | | |  | | | | | Individual | |  |  | |
| **Other non-health outcomes^[[9]](#footnote-9)^** | 1) | | | | | | | | | | | | | 3) | | | | | | |
|  | 2) | | | | | | | | | | | | | 4) | | | | | | |
| **Costs** | Health sector | | | | |  | | | *Details* | | | | | | | | | | | |
|  | Non-health sector | | | | |  | | | *Details* | | | | | | | | | | | |
| **Main results^[[10]](#footnote-10)^** |  | | | | | | | | | | | | | | | | | | | |
| **Additional notes^[[11]](#footnote-11)^** |  | | | | | | | | | | | | | | | | | | | |

1. E.g. Return on Investment (ROI) [↑](#footnote-ref-1)
2. How does change in food intake affect nutrition? E.g. how effect on vitamin A deficiency was estimated [↑](#footnote-ref-2)
3. E.g. analysed using a Difference-in-Difference approach [↑](#footnote-ref-3)
4. E.g. assumptions or surveys [↑](#footnote-ref-4)
5. E.g. time horizon [↑](#footnote-ref-5)
6. E.g. specific methods or assumptions used to convert treatment effect into DALYs [↑](#footnote-ref-6)
7. E.g. case averted [↑](#footnote-ref-7)
8. How to estimate differential costs and effects of the intervention [↑](#footnote-ref-8)
9. Cross-sectoral impacts on outcomes that might be relevant from other non-health care perspectives [↑](#footnote-ref-9)
10. E.g. cost per DALY [↑](#footnote-ref-10)
11. E.g. details about modelling [↑](#footnote-ref-11)
